# Frey’s syndrome as a differential diagnosis for food allergy: a case report

**DOI:** 10.3399/bjgpopen17X100653

**Published:** 2017-02-15

**Authors:** Nia Williams, Nandinee Patel, Abbas Khakoo

**Affiliations:** 1 Paediatric Registrar, Paediatric Department, Hillingdon Hospital, London, UK; 2 Paediatric Registrar, Paediatric Department, Hillingdon Hospital, London, UK; 3 Consultant Paediatrician, Paediatric Department, Hillingdon Hospital, London, UK

## Introduction

Frey’s syndrome, or auricotemporal syndrome leads to facial flushing and a rash associated with eating certain foods.^1,2^ It is caused by injury to the auriculotemporal nerve, whether by trauma or following surgery to or around the nerve.^1–4^ This case describes a child who was referred by their GP to a paediatric allergy specialist and was later diagnosed with Frey’s syndrome.

## Case report

A 5-year-old girl presented to her GP with a history of an erythematous rash that appeared on her left cheek associated with eating certain foods including strawberries, apples, and sweets. The rash would appear immediately on mastication and would entirely disappear within 30 minutes of ingestion.

Her medical history was unremarkable apart from a road traffic accident at 3 years of age when she suffered facial and chest trauma leading to a mandibular fracture and right lower lobe collapse. The patient required intubation and ventilation for 9 days on the paediatric intensive care unit and underwent maxillofacial surgery for the mandibular fracture.

**Figure 1.**
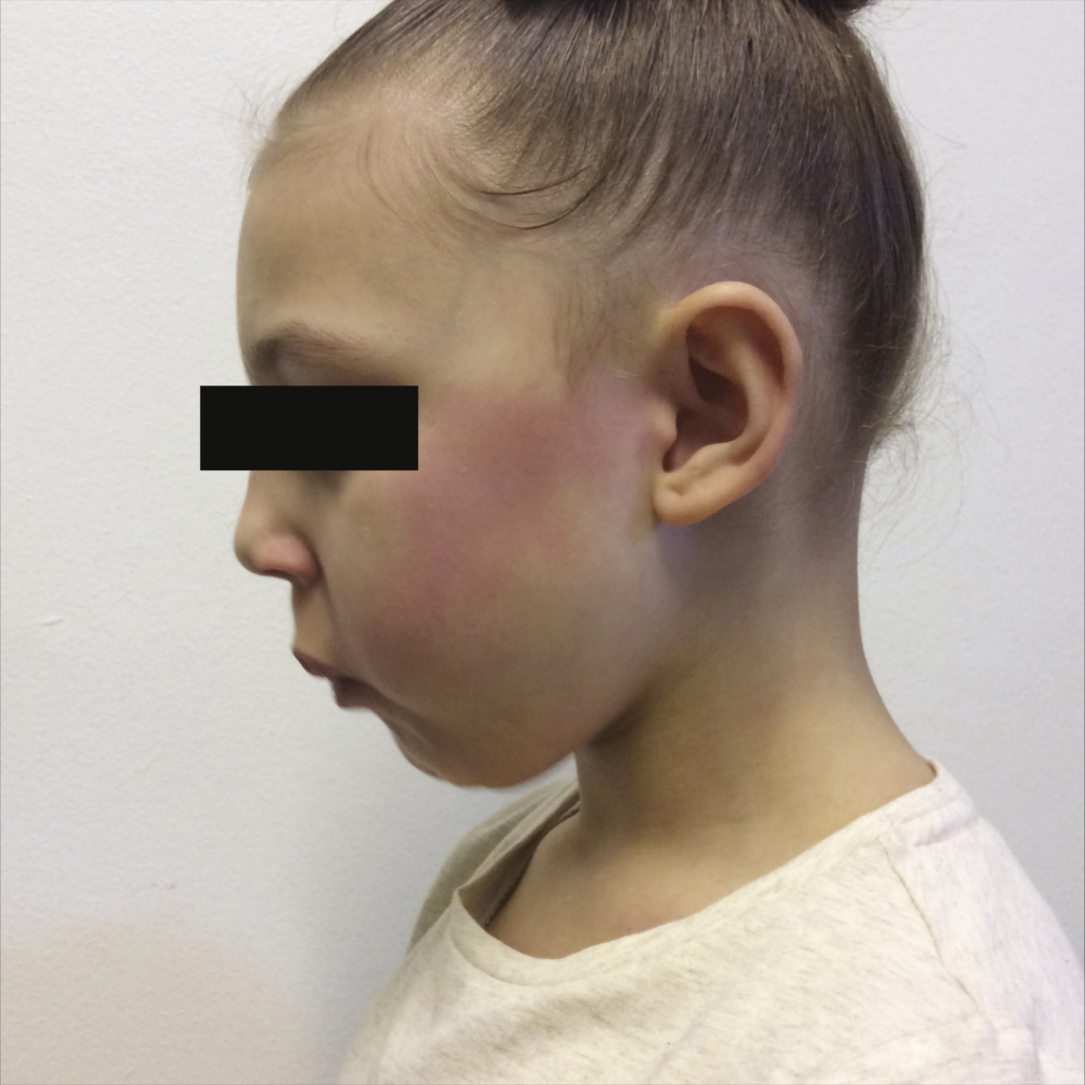

Physical examination revealed a well-grown child with no systemic abnormalities or eczema. Within a few seconds of eating candy a facial flushing appeared on her left cheek, stretching from her the temporal region to the corner of her mouth. This faded within a few minutes as demonstrated in Figure 1. There was no associated lip or tongue swelling or difficulty in breathing.

The patient was referred for skin prick testing which was performed on an extended panel and was negative. She was reviewed by a consultant paediatrician who made a diagnosis of Frey’s syndrome and counselled the family with regard to the non-allergic pathogenesis of this condition.

## Discussion

This case illustrates an interesting presentation of facial rash associated with food and mastication. It is commonly misdiagnosed as food allergy and many children are referred for allergy testing or advised to unnecessarily avoid certain foods until the correct diagnosis is made.^5,6^

Food allergy can manifest as an acute urticarial rash which is characterised by erythematous, raised lesions which are pruritic and may be accompanied by local angioedema.^7^ The rash usually presents within hours of ingestion of the allergen. It would be unusual for allergy to present as a unilateral rash in the same location in response to a multitude of triggers.

Frey’s syndrome, which is also called auriculotemporal syndrome or gustatory flushing was first described by Dupenix in 1757^1^ and was subsequently explained by a neurologist Dr Lucja Frey.^2^ It is caused by injury to the auriculotemporal nerve, whether by trauma or following surgery to or around the nerve. It can present several weeks to years after the injury. Delivery via forceps is a reported cause in children as is facial trauma.^3,4^

On mastication, it immediately manifests as hemifacial flushing and sweating on the cheek, temporal and retroauricular regions. It is triggered by strong tasting foods that increase salivation, such as candies and fruits.^4^ The exact pathology is not well understood but it has been hypothesised that abnormal nerve regeneration occurs after simultaneous damage to the sympathetic and parasympathetic nerves located near the parotid gland and this results in an abnormal autonomic response.^8^

Treatment is not usually necessary as the condition is benign but some patients suffer from social and psychological distress due to the intense facial flushing and may seek treatment. Botulinum toxin injections have been shown to give temporary symptomatic relief.^9^

## Conclusion

This case highlights the need to keep an open mind when assessing patients who present with presumed food allergy. Making a diagnosis of Frey’s syndrome could prevent unnecessary allergy testing and avoid referral to specialist services. The diagnosis can be made on the basis of a detailed clinical history, food diary, and examination of the reported reaction.
